# Complex Composite Odontoma

**DOI:** 10.5005/jp-journals-10005-1066

**Published:** 2010-08-17

**Authors:** Parimala Tyagi, Shilpy Singla

**Affiliations:** 1 Professor and Head, Department of Pedodontics and Preventive Dentistry, People’s Dental Academy, Bhopal, Madhya Pradesh India; 2 Senior Lecturer, Department of Pedodontics and Preventive Dentistry, People’s Dental Academy, Bhopal, Madhya Pradesh India

**Keywords:** Complex, calcified, nonaggressive.

## Abstract

Odontomas are hamartomas composed of various dental tissues, i.e. enamel, dentin, cementum and sometimes pulp. They are slow-growing, benign tumors showing nonaggressive behavior. Most of the odontomes are asymptomatic with unknown etiology, although occasional signs and symptoms related to their presence do occur. Presented here is the case report of 10-year-old girl with impacted left central incisor.

## INTRODUCTION

Odontomas are hamartomas composed of various dental tissues, i.e. enamel, dentin, cementum and sometimes pulp. They are slow-growing, benign tumors showing nonaggressive behavior.^1^ They are classified as complex, when the calcified tissues present simply as an irregular mass composed mainly of mature tubular dentin, or compound, if there is superficial anatomic similarity to even rudimentary teeth.^2^ Complex odontomas are less common than the compound variety in the ratio 1: 2.^3^

The etiology of odontomas is unknown, although local trauma, infection, and genetic factors have been suggested. One aspect of the etiology of odontomas is most result from extraneous buds of odontogenic epithelial cells.^4^ Most of the odontomes are asymptomatic, although occasional signs and symptoms related to their presence do occur. They generally are unerupted or impacted teeth, retained deciduous teeth, swelling and evidence of infection.

## CASE REPORT

**Fig. 1:**
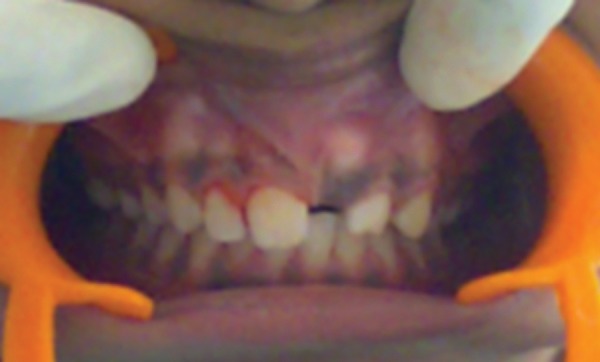
Intraoral photograph showing unerupted tooth with sufficient space for eruption

A 10-year-girl was referred to the Department of Pedodon-tics and Preventive Dentistry due to the failure of the left maxillary central incisor to erupt. Past family and medical histories were unremarkable. There was no history of trauma, deformations, or swelling of the maxillofacial region. Intraoral examination revealed normal colored mucosa, increased in volume of the ridge and absence of maxillary left central incisor. The space was sufficient for eruption of the tooth (Fig. 1).

**Fig. 2:**
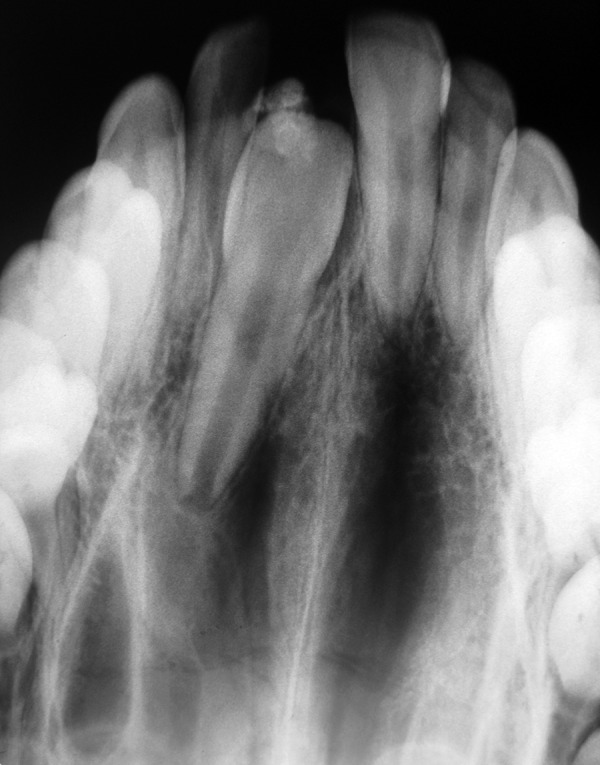
Maxillary occlusal view showing calcified mass

**Fig. 3:**
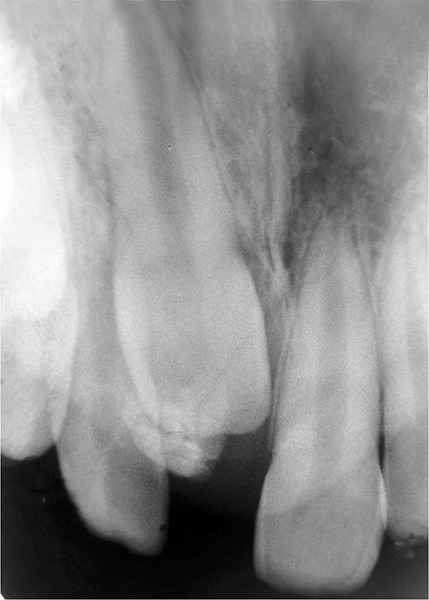
Periapical radiograph showing unerupted maxillary left central incisor with fully formed roots

An intraoral periapical radiograph (Figs 2 and 3) showed an unerupted maxillary left central incisor in the correct vertical position and well-developed but covered with a round opaque calcified mass. Based on the clinical and radiographic evaluation, the diagnosis of a complex odontoma associated with the tooth was established.

**Fig. 4:**
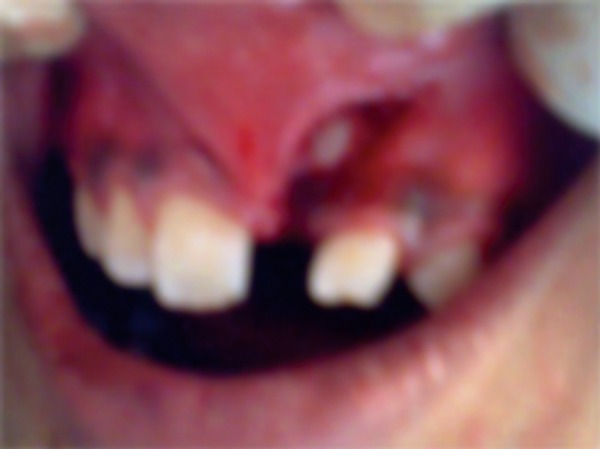
Surgically exposed central incisor after removal of odontome

**Fig. 5:**
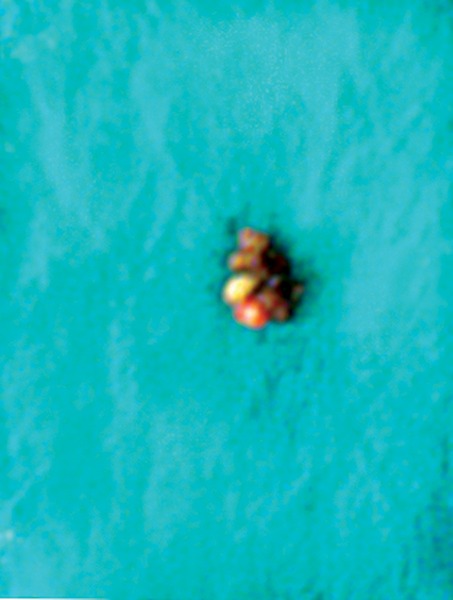
Calcified mass

Surgical removal of the mass was accomplished under local anesthesia. A full thickness mucoperiosteal flap was reflected. A thin layer of the bone overlying the labial surface was removed and the calcified mass was exposed (Fig. 4). There was obliterated calcified mass which was obstructing the eruption of central incisor (Fig. 5). The flap was replaced and secured with 3-0 silk sutures.

**Fig. 6:**
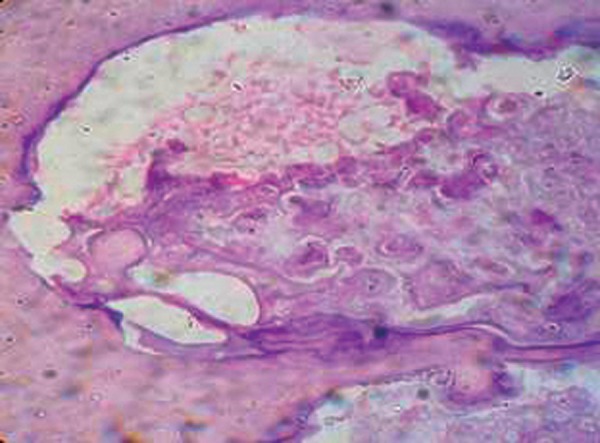
Histopathogical picture of complex odontoma

**Fig. 7:**
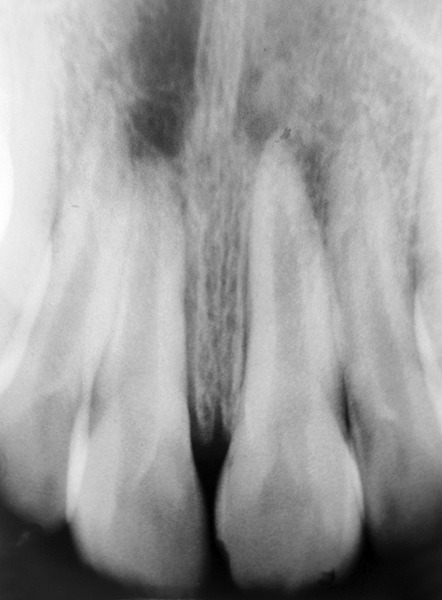
Postoperative periapical radiograph after 6 months showing erupted permanent central incisor

Histopathologic examination (Fig. 6) after decalcification by 10 percent formic acid revealed irregular areas of dental tissues with lack of morphodifferentiation. The section showed areas of dentin in haphazard arrangement of calcified dentin with regular dentinal tubules and cementum like basophilic tissue in globules. The calcified tissue also represented some areas of enamel spaces, and delicate fibrocellular pulp tissue. The tissue was confirmed histo-pathologically as complex composite odontoma.

The postoperative period was uneventful (Fig. 7). The patient is being monitored at regular intervals. The tooth erupted in the right position without orthodontic intervention.

## DISCUSSION

Although, the majority of unerupted teeth are seen in the permanent dentition, it is relatively common in the early-mixed dentition. It has been suggested the possible reasons for failure of eruption may be a lack of space, malformation from early trauma, and mechanical obstruction due to such conditions as a supernumerary tooth, an odontoma, or scar tissue due to early loss of primary teeth.^5-7^ Odontomas often cause disturbances in the eruption of teeth such as, impaction or delayed eruption, retention of primary teeth, or abnormalities in the position of the teeth such as tipping or displacement of adjacent teeth.^5-8^ In this case, the reason of uneruption of the permanent maxillary left central incisor was the presence of a complex odontoma. Complex odontomas are usually located in^9,10^ the first and second molar area of the mandible. A slight majority of odontomas are localized on the right side of the mandible compared to the left. Compound odontomas are approximately twice as common as complex odontomas, and more of the former occurs in the incisor and canine areas of the maxilla.^9,10^ However, the present complex odontoma was associated with an impacted maxillary left central incisor. This localization was regarded as rare.

Most odontomas are detected during the first two decades of life, and the mean age at the time of diagnosis is 14 years.^11,12^ Although, the compound type variety is approximately equally distributed between the genders, 60% of complex odontomas occur in women.^2,9^ Compound odontomas seldom cause bony expansion, but complex odontomas often cause slight or even marked bony expansion.^2,12^ The age, gender, and slight bony expansion in this case are in accordance with previous reports. The lesion should be surgically removed and the specimen should be carefully examined microscopically to rule out ameloblastic odontoma or myxofibrous hyperplasia.^10^ Kramer et al mentioned when a tooth fails to erupt, the follicle may become thickened and it may have an appearance similar to that of an odontogenic fibroma or myxoma.^13^

In our case study, we present a mature complex odontoma, which should be differentiated from cementoblastoma, osteoid osteoma and fibro-osseous lesions, such as cemento-ossifying fibroma. A cementoblastoma presents as a well-defined radiopaque mass attached to the tooth root and surrounded by a radiolucent rim.^2,5^ Osteoid osteomas are characterized by a small ovoid or round radiolucent area surrounded by a rim of sclerotic bone; the central radiolucency exhibits some calcification. Cemento-ossifying fibroma presents as a well-defined radiolucency with increasing flecks of calcification as it matures; it is not surrounded by a radiolucent rim and it is diffuse with normal bone.^14^ Also, none of these is associated with an impacted tooth.

The odontoma presents as a well-defined radiopacity situated in bone, but with a density that is greater than bone and equal to or greater than that of a tooth. It contains foci of variable density. A radiolucent halo, typically surrounded by a thin sclerotic line, surrounds the radiopacity. The radio-lucent zone is the connective tissue capsule of a normal tooth follicle. The thin sclerotic line resembles the corticated border seen in a normal tooth crypt. The developmental stages can be identified based on radiologic features and the degree of calcification of the lesion at the time of diagnosis.^15^ The first stage is characterized by radiolucency due to the absence of dental tissue calcification, the second or intermediate stage shows partial calcification and the third or classically radiopaque stage exhibits predominant tissue calcification with the surrounding radiolucent halo described above.

Surgical exposure and elimination of mechanical obstruction is frequently the treatment of choice and spontaneous eruption can then be expected.^16^ Since the occurrence of a complex odontoma in this particular location is rare, removal of the mass overlying the tooth led to the eruption of the permanent incisor in its position.

Early diagnosis of odontomas is important for preventing craniofacial and tooth developmental problems. The early diagnosis accompanied by a proper treatment at the right time will result in a favorable prognosis. In order to diagnose developmental abnormalities as soon as possible, a professional team of pediatric dentists should be aware of the importance of clinical and radiographic examinations.
